# Gamifying water crisis management: A serious game for drinking water contamination emergency response

**DOI:** 10.1371/journal.pone.0321210

**Published:** 2025-04-01

**Authors:** Jingyan Huang, Catherine M. Ashcraft, Weiwei Mo

**Affiliations:** 1 Natural Resources & Earth Systems Science Program, University of New Hampshire, Durham, New Hampshire, United States of America; 2 Department of Civil and Environmental Engineering, University of New Hampshire, Durham, New Hampshire, United States of America; 3 Department of Natural Resources and the Environment, University of New Hampshire, Durham, New Hampshire, United States of America; Radford University Artis College of Science and Technology, UNITED STATES OF AMERICA

## Abstract

This paper presents a serious game that simulates a water crisis triggered by the spill of an unregulated chemical. The game includes five stakeholder roles representing a chemical manufacturer, resident, water treatment plant, environmental agency, and health department, in addition to a facilitator role. The game seeks to provide players with practical experience of the communication and collaboration needed among different stakeholders to prepare for and respond to water contamination emergencies. Initial findings from game sessions with 41 participants suggest that frequent, proactive, and transparent communication can expedite the decision-making process and resolve the crisis more effectively. The game results also reveal challenges in inter-organizational coordination and communication, highlighting the need for training and standardized communication terminologies and protocols.

## Introduction

Despite the significant strides that have been made towards the protection of water safety and public health, drinking water contamination incidents remain common in the United States. The United States Environmental Protection Agency (EPA)’s Safe Drinking Water Information System recorded about 68,996 health-based violations in community water systems between 2014 and 2023 [1]. In addition, over 280,000 chemical release incidents were reported between 2010 and 2019, of which 3,931 posed a direct threat to drinking water sources [2]. When a drinking water contamination incident occurs, it is important to respond effectively to reduce its potential harm. Unfortunately, the landscape of such emergency responses is often marred by confusion [3], miscommunication [4], inefficiencies [5], poor collaboration [6], and scarcity of information [7]. For instance, in the 2014 Elk River chemical spill in West Virginia, the absence of immediate physiochemical and toxicological information of the chemicals spilled hindered the response efforts [8]. The inconsistent health-based screening levels recommended by the Centers for Disease Control and Prevention (CDC), independent toxicologists, and the state of West Virginia further contributed to public confusion and mistrust [9]. In the same year, the Hoosick Falls water contamination event and the Toledo water crisis exhibited a lack of communication and coordination across the local government and the state public health and environmental agencies, leading to delay in actions and escalation of the crises [10,11]. Similarly, the unclear and uncoordinated communication regarding the expected duration and resolution plan in the 2018 Salem, Oregon algal bloom caused panic among residents [12]. It is therefore important to characterize the challenges associated with drinking water emergency response, identify the opportunities to overcome these challenges, and to prepare the workforce for potentially complex emergency situations in the future.

Serious gaming is a suitable tool for this purpose. Serious games (hereafter sometimes referred to as “game”), also known as role play games or tabletop games, are games intended to fulfill a serious purpose, which blends active learning with a purposeful mission to convey ideas and values, and in some cases, to persuade players towards certain viewpoints or actions [13]. In serious games, participants are engaged in fictional yet realistic scenario-based challenges. They offer an engaging and immersive platform, allowing players to experiment with decisions and strategies that would be costly or impractical to test in the real world [14]. Furthermore, serious games provide a conducive environment for discussion, negotiation, learning, innovation, and relationship-building, thereby improving decision-making through enhanced understanding, communication, and creative problem-solving [15,16]. This form of experiential learning has gained significant traction over the past decade, evolving into an educational tool in fields such as military training [17], health care [18], climate change mitigation [19], and urban planning [14,20].

Previous water-related serious games have been primarily focused on water resource planning and allocation [21–25], stormwater management [26–31], and water pricing and affordability [32]. Very few games have been geared towards drinking water emergency responses. The US EPA published a serious game with 16 different natural and man-made drinking water hazard scenarios to help drinking water and wastewater utilities practice, test, and improve their emergency response plans and procedures [33]. However, the game does not incorporate multiple roles to mimic actual communication and coordination barriers across different stakeholders. In addition, none of these games considered a chemical release scenario. Two additional games have been designed for environmental and health crisis management. “Managing the Micronium Mess” is an eight-party negotiation simulation that addresses the risks associated with the production and disposal of Micronium – a synthetic material integral to several industries but harmful to the environment and human health [34]. The game seeks to facilitate developing consensus and effective strategies to manage the impending crisis before it becomes unmanageable. Another game, “Boosting Effectiveness in Water Operators’ Partnerships (BEWOP)”, is a seven-party role play game aiming at solving the water quality risk caused by increased agricultural activities and the development of the industrial sector [35]. The primary focus of this serious game is to proactively prevent chemical contamination associated with the ongoing expansion of agricultural and industrial sectors. To the authors’ knowledge, there is no existing serious game that specifically addresses multi-organizational communication and collaboration in response to acute drinking water contamination events. Moreover, previous games typically provide players with fixed information for gameplay, without a mechanism to introduce new information as the game progresses. This approach fails to mirror real-world scenarios where information continuously emerges and evolves.

The City of Leaf Game directly addresses these gaps by simulating a chemical spill incident and engaging five roles, each representing key stakeholders involved in a drinking water emergency. Unlike previous games, the interactions between players trigger new information, thereby varying the game’s progression and reflecting the fluid nature of real-world situations. By administering this game and analyzing outcomes observed in game sessions, this study seeks to improve players’ readiness to handle water contamination emergencies and explore how inter-organizational communication and collaboration can contribute to more effective drinking water emergency responses. Below, we elaborate on the methodology behind the game’s development, from establishing learning goals and identifying the target audience, to the creation of game settings, participant roles, gameplay rules, and the iterative process of testing and refining the game. We then discuss the outcomes observed and reflections after administering the game to 41 participants.

## Methods

According to Hirumi and Stapletion (2008), the design process for serious games can be divided into four phases: 1) the concept development phase, 2) the pre-production phase, 3) the prototype and production phase, and 4) the post-production phase. This structured framework has been adopted to guide the development of the City of Leaf Game. Each of these steps is detailed below.

### Concept development phase

The concept development phase characterizes the target audience and defines learning goals [36]. The game’s target audience includes drinking water system operators, municipal and state emergency response managers, and graduate and undergraduate students in relevant programs. Serving as an effective training tool, it allows these professionals and future practitioners to test and refine their decision-making strategies within a safe environment and equips them with essential insights and skills for managing real-life emergencies. The learning goals of this game are: 1) to gain firsthand insight into the complexities and challenges associated with drinking water contamination crises, fostering a proactive mindset towards implementing change; 2) to recognize the critical role of communication, negotiation, and collaboration in resolving environmental issues, enhancing the ability to work collectively; and 3) to acquire best practices for addressing water contamination, enabling participants to apply these strategies effectively in real-world scenarios.

### Pre-production phase

The pre-production phase involves the development of the game’s settings, roles, and gameplay rules. This phase typically ends with the completion of a game prototype [36].

#### Game setting.

The core setting of the game revolves around a water crisis triggered by the release of an unregulated and inadequately studied chemical. Instances of contamination involving new chemical substances are an unexpected yet common problem. An example of this is the 2014 Elk River chemical spill in West Virginia, where limited toxicological information was available on crude 4-methylcyclohexanemethanol (MCHM) at the time [37]. Poor communication during this event delayed the response efforts, complicated the situation, and ultimately led to skepticism and mistrust from the public [38,39]. Resolving such an emergency requires communication and coordination across multiple roles to understand and evaluate the issues at hand and act under uncertainty. The City of Leaf Game could equip participants with the knowledge, understanding, and skills for effective response to complex contamination incidents.

The game is set in a fictional city named the City of Leaf with a population of approximately 15,000 people. The city relies on the Bella River, the sole surface water source of the Leaf Drinking Water Treatment Plant. The game unfolds as an unregulated and under-researched chemical leaks from the underground tanks of the SynthoChem Corporation – a chemical manufacturer situated upstream of the Leaf Drinking Water Treatment Plant – into the Bella River. The SynthoChem Corporation plays a vital role in Leaf’s economy, with 20% of the Leaf population being closely connected to the company either through direct employment or by virtue of roles closely intertwined with its operations. The game represents a restricted awareness model. No party has confirmation of a drinking water crisis at the beginning of the game, although residents and SynthoChem manager suspect potential issues. The water treatment plant operator, during routine monitoring, detects a slight increase in Total Organic Carbon (TOC) levels, but no alarms are triggered. As the water crisis unfolds, each player’s objective is to gather information and act upon the situation to resolve the issue in the fewest game rounds, while maximizing their role’s interests. The game involves negotiations surrounding water safety, contaminant source removal, drinking water services, environmental remediation, public health monitoring, and harm compensations, among others.

#### Design of the roles.

To develop the roles for the game, a comprehensive review of the federal, state, and local drinking water emergency planning and response documents, alongside case studies of historical drinking water contamination incidents, was conducted to identify the key parties engaged in drinking water emergency response. As a result of this review, 34 key roles were cataloged, ranging from local response units such as fire and police departments [40,41] to federal agencies including USEPA [42], CDC [43], and FEMA [44,45], as well as various non-governmental organizations [46]. A complete list of the responsibilities and potential response actions related to each of the 34 roles can be found in S1 Table of the supporting information (SI). These 34 roles were then consolidated into 5 roles to allow for a more playable game, reflecting their significance and common functions within the context of a drinking water emergency response. The consolidated roles are detailed in Table 1.

**Table 1:** The five designed roles in the City of Leaf Game.

Roles	Description	Real world roles represented	Real world responsibilities and decisions	Objectives in the designed game	Capabilities & Responsibilities in the designed game
The SynthoChem Corporation	A major chemical manufacturer in the city and the polluter.	Polluter	Liable for costs of containment, cleanup, and damages resulting from the release [47].	Minimize financial losses due to the chemical leakage and preserve the company’s public image.	Responsible to report chemical leakages to the Environmental Agency and handle the leak if the leakage poses sudden threats to public health. Able to conduct leakage investigation and on-site remediation.
Resident in the City of Leaf	A resident affected by the water crisis.	Affected public	File personal injury claim or negligence claim for injuries or damages due to chemical spills [48].	Ensure the tap water is safe and sufficient for personal and family use, while avoid increasing water rates.	Able to request blood test and initiate lawsuits for compensation.
The Leaf Drinking Water Treatment Plant	A local water supplier.	Water Utility, Neighboring Water Utility, Water Vendors	Notify the public of water advisories; distribute emergency water supplies; restore tap water supply [42,49].	Ensure the tap water is safe and sufficient for consumers and maintain consumer confidence.	Responsible for supplying safe water to the city. Able to do water quality test, use emergency on-site treatment, issue do-not-use order and supply bottled water, and adjust water rate.
The Environmental Agency	The regulatory body overseeing environmental compliance.	Local Police Department, State Emergency Management Agency (EMA), State Environmental Protection Agency, State Homeland Security, USEPA	Provide environmental recovery assistance if a contamination incident is confirmed; take enforcement actions against violators of Safe Drinking Water Act (SDWA); conduct investigations to identify sources and impacts of water pollution [42].	Protect the environment and public health.	Responsible for ensuring regulatory compliance and timely remediation of contamination. Able to conduct water test, investigate the polluter, mandate the responsible party to pay for environmental remediation, and enforce fines, with access to emergency funds for addressing contamination.
The Health Department	The agency responsible for public health analysis and technical support.	Local Emergency Medical Care, Local Department of Health, Local Poison Control Center, State Department of Health, US Department of Health and Human Services (DHHS), Agency for Toxic Substances and Disease Registry (ATSDR), CDC, Environmental and Public Health Laboratories	Provide analytical support in the case of a suspected contamination incident; assist in diagnosing and investigating disease outbreaks; set up disease surveillance system; issue health-related public notifications to protect public health from contamination [45].	Safeguard long-term public health.	Responsible for health risk evaluation and public communication, Able to conduct toxicity analysis to establish chemical’s Health Advisory level and assess its potential health effects.

#### Gameplay.

Participants of the City of Leaf Game receive two sets of instructions: general instructions and confidential instructions. General instructions provide common knowledge to all roles, outlining the game’s setting and explaining the gameplay mechanics. Confidential instructions provide private information exclusive to each role, including a one-page narrative describing the backstory of the role that contains their distinct responsibilities, knowledge, and capabilities, a one-page worksheet that specifies their primary concerns and suggested strategies, and a one-page note-taking sheet that facilitates recording communications, costs incurred, and voting outcomes in each round. In addition, each role also receives a set of action cards, which details potential actions they can take to fulfill their responsibilities or address specific concerns, as well as virtual “checks” that allow for the transfer of funds between roles or payment of expenses associated with certain actions.

**Fig 1:**
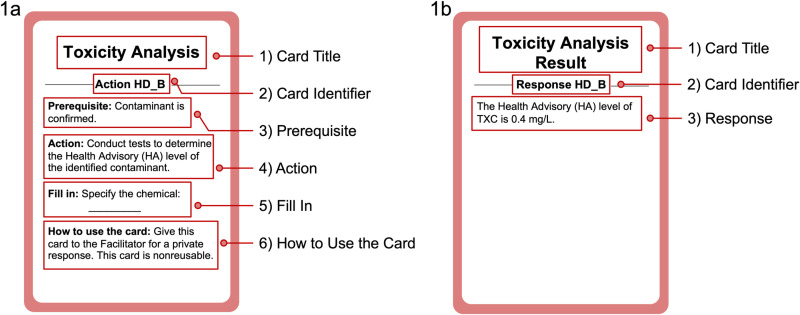
A sample action card (1a) and its corresponding response card (1b). The action card includes card title, card identifier information, prerequisite to use the card, the specific action to be taken, the required information for the players to fill in before using the card, as well as instructions regarding how to use the card. The response card includes card title, card identifier information, and the specific response related to the initiated action. Action cards are initially distributed to the players, while the response cards are initially with the facilitators.

The game is structured into rounds. Each round consists of three phases:

- ***Spread out:*** The “spread out” phase provides participants 5 minutes to spread out and engage in private, one-on-one conversations, with the option to speak to multiple roles if time allows. The information asymmetry between roles necessitates communication. Their different and sometimes conflicting interests require them to negotiate and collaborate. They need to decide which information to share or withhold, whom to approach for information, and who might benefit from their information. As the game progresses, new information can emerge, altering their understanding of the current condition and response strategies. Key information exchanges between roles that influence the progression and resolution of the crisis scenario within the game, include the awareness of contamination, contaminant identification, contaminant treatment standard, and contaminant concentration changes over time. S2 Table in the SI provides an overview of how each piece of key information originates, is disseminated, and affects the game’s outcome.
- ***Gather:*** The “gather” phase allows each player to execute up to two role-specific action cards. These cards enable a range of activities, such as conducting investigations to acquire additional information, mitigating the impact of incidents, and requesting specific actions from others. When executing an action card, players may be instructed to give the card to the facilitator in exchange for a response card, which contains additional essential information for solving the water crisis at hand. To facilitate the card exchange process, both action cards and response cards are color-coded by role. Fig 1 provides a sample action card and the corresponding response card. An action card may contain up to 6 distinct sections. “Card title” briefly summarizes the action. “Card identifier” is a code that aids in matching an action card with its corresponding response card. “Prerequisite” lists the conditions that must be met before the card can be activated. “Action” specifies the activities to be undertaken. “Fill in” has required blanks that players must fill in before using the card. “How to use the card” guides players on who to give this card to, whether the action is conducted privately or announced publicly, and whether the card is reusable. In addition to these regular action cards, each role also has a public announcement card and a free action card. The public announcement card allows them to make a public announcement to all players once. The free action card allows participants to initiate an action that falls outside the predefined options listed on the regular action cards to encourage creative problem solving.

- ***Vote***: In the “vote” phase, the facilitator initiates a vote, and players cast their ballots based on their satisfaction with their current condition. Any “no” votes extend the game to the next round; a unanimous “yes” vote ends the game, indicating that the crisis has been thoroughly resolved. This game-ending criterion encourages players to consider multifaceted solutions that go beyond just technical fixes of water contamination but also broader social, economic, and environmental factors. Reaching unanimity in fewer rounds is deemed a more effective resolution, demonstrating the players’ ability to quickly coordinate the interests of all stakeholders and implement comprehensive solutions that effectively address the entire scope of the crisis.

Guided by the facilitator, participants transition between rounds and move through the three phases within each round. After the game, the facilitator leads a debriefing session to encourage players from each role to evaluate their success in achieving goals, discuss strategies employed, challenges faced, and the methods used to overcome them, and explore opportunities for improving inter-organizational communication and collaboration. The game design also includes a detailed instruction packet for the facilitators, which includes the response cards and the potential debriefing questions for discussion. The complete materials for conducting the simulation, including teaching notes, general and confidential instructions for players, and facilitator instructions, can be accessed online [50].

### Prototype and production phase

**Fig 2:**
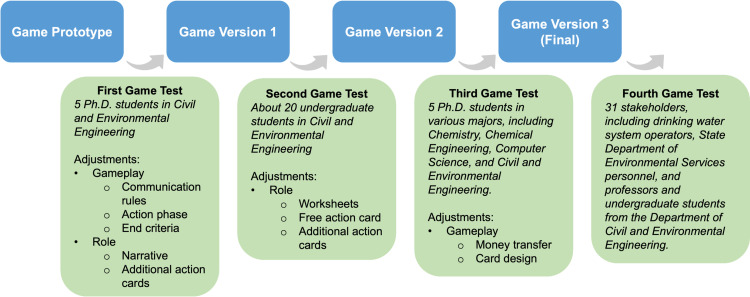
Iterative Game Development Process. This flowchart details the iterative development of the game from an initial prototype to its final version through four test phases. Each phase involves a distinct group of testers who provide feedback leading to specific adjustments in gameplay and role, such as communication rules, action card design, and money transfer mechanisms.

The prototype and production phase transform the initial prototype into the final product. During this phase, the preliminary gameplay mechanics embedded in the prototype are tested, evaluated, and refined through iterative feedback and a modification cycle [36]. To improve the design, the game was tested in four rounds involving graduate and undergraduate students from diverse majors and real-world stakeholders, providing a broad range of perspectives and feedback (Fig 2).

The first game test involved five Ph.D. students in the environmental engineering program, with their feedback gathered during a post-game discussion. A significant change at this stage was the rules governing role communication. In the original game design, a predetermined order of communication was written into the game rules, and players could only communicate one-on-one in private. While two people were communicating, other players had to wait. Although each conversation was limited to 1 minute, our testing showed this approach to be time-consuming and less engaging. Furthermore, some of the roles were not engaged in the conversation until several rounds had passed. The updated design allowed all roles to initiate conversations from the start, permitting simultaneous discussions to maintain engagement. Additionally, adjustments were made to consolidate the action cards execution phase, allowing for both private and public actions. A change was also made to the mechanism of ending the game to strengthen player engagement, which went from an action-based ending rule (e.g., the game ends when the appropriate actions are taken by the Leaf Drinking Water Treatment Plant in restoring water service) to a unanimity-based ending rule. Additionally, the narratives of the roles were refined for clarity, and new action cards were added to expand each role’s capabilities.

The second test involved three groups of undergraduate students majoring in civil and environmental engineering. Feedback was gathered using a Qualtrics survey (see S1 Appendix), focusing on gameplay experience, the game’s realism, playability, and flow. In addition, three facilitators convened post-game testing to reflect on their observations and discuss the potential for future revisions. Feedback indicated that students struggled to distill the key information provided in the confidential instructions of their roles. To address this, a worksheet for each role was introduced, outlining primary concerns and suggesting strategies to help participants understand their responsibilities and guide decision-making. Another significant adjustment was the introduction of free action cards. The free action cards allow players the flexibility to propose and execute creative and adaptive actions. Minor adjustments were also made to the roles by adding more action cards.

The third game test involved five Ph.D. students in various majors, including chemistry, chemical engineering, computer science, and civil and environmental engineering. Their feedback was gathered during a post-game discussion. In this phase, two significant changes were made. We introduced virtual checks to replace verbal statement expenditures and costs to avoid errors and confusion in financial tracking and enhance game immersion. In addition, we redesigned the action cards to feature clear titles for quick recognition, reducing the need for players to repeatedly read detailed text and thus ensuring that players could focus more on strategic decisions rather than navigating cumbersome text, enhancing the overall flow and enjoyment of the game.

The fourth game test was deployed at a conference across five groups with 31 real-world stakeholders. Post-game, a survey was distributed to gather participant feedback on the educational value of the game, its degree of realism, and suggestions for enhancements in its design and administration. Out of the 31 participants, 13 completed the surveys. All respondents found the game to be at least “slightly helpful”, with 9 out of 13 reporting it as “very helpful”. One participant, reflecting on personal experience with a similar situation, commented on the game’s realism, noted that “having experienced a similar situation in my town of the contamination issue and figuring out how to deal with it on the fly was very realistic.” In summary, the game was perceived as beneficial in improving participants’ readiness and response capabilities regarding water contamination issues. The realistic simulation of crisis scenarios and the practical skills delivered through the gameplay were particularly highlighted as strengths of the game. The feedback underscored the game’s potential to provide real-world benefits.

### Post-production phase

The finalized game was administered to seven groups of civil and environmental engineering undergraduate students, totaling 41 participants to investigate the impact of communication strategies on the effectiveness of emergency response in a simulated drinking water contamination crisis. The project was reviewed and approved by the University of New Hampshire Institutional Review Board (IRB) for the Protection of Human Subjects in Research under IRB # FY2022-201. The recruitment period for this study was from February 19, 2024, to February 29, 2024. Participants signed the consent forms before participating in the research project. During these game sessions, participants recorded their communications, actions, expenditures, and satisfaction following each round. After each game session, a debriefing session was conducted, allowing participants to share their personal game experiences and their perspectives of different roles. The debriefing session was recorded and used to identify challenges in inter-organizational communication and collaboration during a water crisis.

## Results and discussion

Below we detail our results and discussion from the seven groups that participated in the post-production phase in four aspects: 1) communication frequency, 2) communication transparency, 3) terminology divergence, and 4) clarity in roles and responsibilities.

### Communication frequency

**Fig 3:**
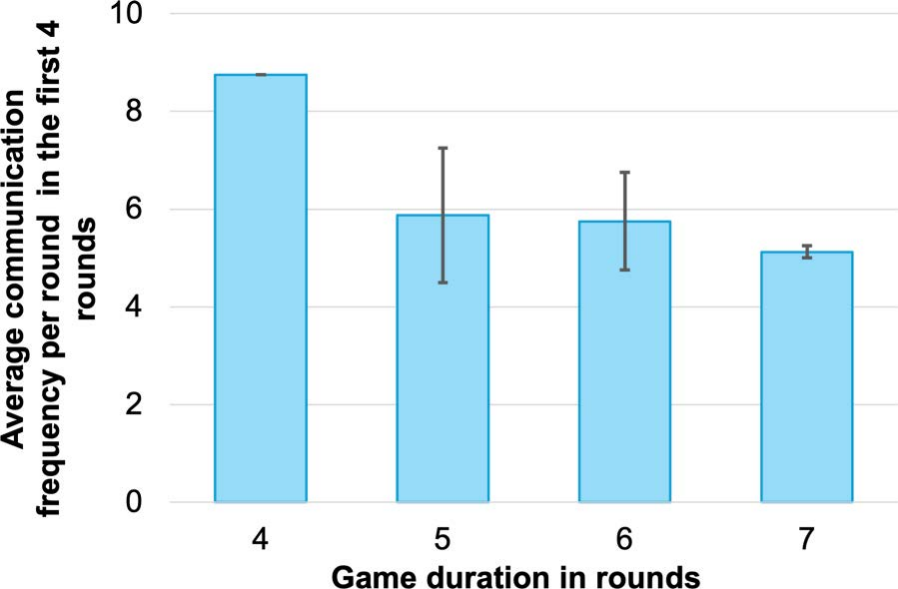
Average Communication Frequency per Round in the First Four Rounds by Game Duration. The average communication frequency per round of all groups with the same game duration is shown in the box plot. The error bars show each group’s actual average communication frequency per round of the first four rounds.

The seven groups that participated in the post-production game administration completed the game in 4 to 7 rounds. Specifically, one group completed in 4 rounds, two in 5 rounds, two in 6 rounds, and two in 7 rounds. Our recorded communication patterns of the seven groups show that there is a notable correlation between the communication frequency, especially in the early rounds of the game, and the total number of rounds the groups took to complete the entire game. Fig 3 presents the average communication frequency per round in the first four rounds of the game, grouped by game duration. Here we calculated the average communication frequency per round by dividing the sum of the bi-directional communication links in the first four rounds by four. Each communication link indicates a conversation between two roles. The findings revealed a trend where groups with higher communication frequencies in the early stages tend to resolve the simulated water contamination crisis in fewer rounds. This indicates the importance of frequent communication among the stakeholders in effectively resolving a water contamination crisis.

**Fig 4:**
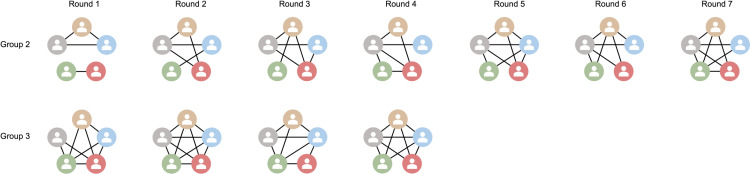
Communication Patterns in Each Round for Groups A and B. Group A (top) finished the game in seven rounds, while Group **B** (bottom) finished the game in four rounds. Each line indicates a bi-directional conversation between two roles. The roles are color coded as follows: SynthoChem Corporation - grey, Resident in the City of Leaf - brown, Leaf Drinking Water Treatment Plant - blue, Environmental Agency - green, and Health Department - red.

We took a deeper dive into the communication patterns of the groups, using two groups as an example (Fig 4). Group A is one of the two groups that completed the game in seven rounds, while Group B completed the game in four rounds. Group A began with a sparse communication pattern, where players primarily interacted with only one or two others. This limited connectivity in the early stages likely restricted the flow of crucial information to other stakeholders, which hindered a holistic understanding of the crisis, thereby delaying effective decision-making and resolution. As the game progressed, interactions within Group A increased, but the initial rounds’ restricted communication likely set the tone for slower overall progress. Group B, in contrast, had a more interconnected communication pattern from the beginning. Each role engaged with at least two others consistently, forming a denser network of communication. The consistent and extensive interactions among roles within Group B ensured that each player was well-informed and actively participating in decision-making processes. It did not merely speed up the crisis resolution but also potentially contributed to more well-rounded solutions, as multiple perspectives were often integrated into decision-making. Findings from this analysis suggest that the establishment of robust communication channels and protocols early in crisis management is crucial for effective and timely crisis resolution.

### Transparency in crisis communication

While communication frequency reveals the players’ intention or willingness to exchange information, effective emergency response also relies on the content of the information being shared. Our findings show that transparency and openness to sharing about the actual situation led to more effective responses. During post-game discussions, the player of SynthoChem Corporation from Group B highlighted the proactive steps taken to resolve the crisis, including immediate reporting of the leakage to both the Environmental Agency and the Health Department. The SynthoChem Corporation players from two other groups made a public announcement about the chemical leakage in the initial round of the game. In the post-game discussion, other players from these two groups expressed their appreciation for the immediate and transparent sharing of critical information, which informed the decision making of other players in the game. Participants expressed in the post-game discussion that transparency in communication allowed all roles to have a clear understanding of the situation, fostering trust and collaboration. It also ensured that critical decisions were based on the most comprehensive information available. In contrast, the SynthoChem Corporation in Group A withheld information during the game when asked about potential issues. The initial hesitancy to disclose information had cascading effects, worsening the situation and complicating response efforts. The post-game discussion also revealed that withholding information resulted in skepticism and mistrust, which hindered cooperation during response efforts. Our findings indicate the need for clear policies and guidance regarding the disclosure of critical information during emergencies. Such policies should define what constitutes necessary transparency and set standards for timely and accurate communication. In addition, it is important to prepare and train stakeholders for effective and transparent communication during emergency conditions.

### Terminology divergence in crisis collaboration

Our observations of the games’ progressions underscore the importance of terminology consistency and shared understanding in facilitating inter-organizational collaboration. For instance, multiple groups experienced a communication barrier between the Health Department and the Drinking Water Treatment Plant. The Health Department determined the Health Advisory (HA) level for the contaminant – indicative of the concentration at which no known or anticipated adverse health effects were likely, with a significant margin of safety. This HA level, when no formal Maximum Contaminant Level (MCL) is established for a new or unregulated contaminant, served as a critical communicative benchmark. However, while the Health Department communicated its findings using the term “HA level”, the Leaf Drinking Water Treatment Plant operated with the term “MCL” to guide their treatment processes and ensure regulatory compliance. This difference in terminology led to confusion at the Leaf Drinking Water Treatment Plant in certain groups, as they were familiar only with MCL and initially struggled to fully grasp the implications of the HA level provided by the Health Department. This discrepancy temporarily delayed their ability to promptly adjust treatment processes to align with the safety level suggested by the Health Department.

In addition, the Environmental Agency and SynthoChem Corporation in several groups encountered misunderstandings due to differing interpretations of the term “remediation”. The Environmental Agency’s request for SynthoChem to pay for environmental remediation was intended to address broader ecosystem and long-term public health concerns through comprehensive actions. However, in some groups, SynthoChem’s understanding of remediation was limited to on-site actions, specifically removing leaking tanks and cleaning up the immediately affected area. This discrepancy in the scope of “remediation” led to misaligned expectations, reflecting a common real-world issue in environmental management where the lack of standardized definitions impedes effective collaboration. Such scenarios illustrate the critical need for implementing common terminology that transcends disciplinary boundaries to ensure that all stakeholders have a clear and shared understanding of the terms and concepts being used. According to the National Incident Management System (NIMS), the use of plain language in emergency response is not just a procedural preference but a public safety imperative, particularly for the safety of first responders and those affected by the incident [51,52]. The Incident Command System (ICS) mandates the use of common terminology and discourages the use of agency-specific codes or jargon, which can lead to communication barriers and hinder effective response in crisis situations [53]. Daily communication habits are crucial, as they shape how responders will communicate under stress. Habitual use of jargon or coded language, for instance, can cause responders to default to such codes in high-stress crisis situations, potentially leading to critical delays or errors [54]. Therefore, it is essential to train first responders to use plain language consistently. Eliminating coded language in routine and emergency communications could enhance interoperability among various agencies and stakeholders, facilitating more effective and precise communication.

### Clarity in roles and responsibilities

Ambiguity in roles and responsibilities significantly hindered the effectiveness of emergency response in the game. Several participants from the Leaf Drinking Water Treatment Plant faced uncertainty in deciding whether to implement emergency on-site treatment and continue supplying tap water or to issue a do-not-use order and provide bottled water. This challenge was primarily due to a lack of knowledge about the chemical’s MCL and uncertainty regarding the appropriate contacts for further guidance. This situation highlights the necessity for clearly defined roles and accessible information about each role’s capabilities and responsibilities. In contrast, players of the Health Department frequently noted in post-game discussions that their instructions included the possibility of contacting the Environmental Agency to secure the necessary funds to support some of their actions. This provision helped them identify who to approach for funding when they wanted to start a blood test program but lacked the financial resources. Similarly, the Environmental Department was aware from the beginning that their primary focus should be on containing the source of contamination, as stated in their instructions. This clarity in responsibilities led to consistent actions across all groups, with the Environmental Department prioritizing source removal from the start. These examples from the game illustrate how well-defined roles can enhance the efficiency and decisiveness of response efforts in emergency situations. A clear description of responsibilities equipped each responder with an understanding of their peers’ roles and the organizational hierarchy and communication networks needed for specific information exchange and collaborative efforts. Moreover, when each member of an emergency response team has a definitive understanding of their individual duties and the expectations placed upon them, it can instill a sense of confidence and accountability [55]. Effective emergency response can be improved through training and the creation and upkeep of clear, comprehensive documentation. This documentation, which details the responsibilities of various agencies and personnel, should be easily accessible and simple to understand.

## Conclusions

This paper introduces the design of a serious game that simulates the inter-organizational communication and decision-making of a drinking water contamination crisis resulting from the spill of an unregulated chemical. The aim was to highlight the essential role of effective communication between diverse stakeholders involved in such incidents, and to understand how to enhance emergency response performance through more effective inter-organizational communication. Five roles were identified based on a review of the federal, state, and local drinking water emergency planning and response documents alongside case studies of historical drinking water contamination incidents. Each role enters the game with distinct knowledge, capabilities, and goals. Players of the five roles are required to communicate and coordinate to resolve the crisis in the fewest possible game rounds. These dynamics foster a cooperative environment where even though each player may start with self-oriented objectives, the interdependencies created by the crisis necessitate and facilitate a more collaborative approach.

The finalized game was administered to seven groups of civil and environmental engineering undergraduate students, totaling 41 participants to investigate the impact of communication strategies on the effectiveness of emergency response in a simulated drinking water contamination crisis. Our findings show that frequent communication in the early stages and transparent communication practices are linked with more effective and timely crisis resolution. Challenges in inter-organizational collaboration were observed, which underscored the necessity for a common language and a clear understanding of roles and responsibilities among stakeholders from different sectors. This insight points to the value of enhanced training programs that focus on building communication skills, the development and implementation of standardized terminologies and protocols that bridge the communication gap among diverse stakeholders in a crisis, and the development of guidance or policies for timely information disclosure.

While our study provides valuable insights into the potential of serious games as a training tool for complex water crisis management, real-world incidents may involve a broader array of unpredictable elements such as varying environmental conditions, unforeseen political or social responses, and the dynamic nature of public perception and media influence. Future work in this area could potentially expand this game to incorporate a broader range of scenarios or uncertainties into the game design. The game can also be potentially expanded to include additional crisis scenarios such as including infrastructure failures, natural disasters, and more, each presenting unique challenges and requiring specialized response strategies. In addition, the current method of evaluating game outcomes based on duration, while straightforward, may not fully reflect the quality of the decisions made. Future evaluations could consider a broader range of factors, such as the environmental impact of the incident, the overall public health impact, and the economic costs of response efforts.

## Supporting information

S1 Table Stakeholders in Drinking Water Contamination Emergency Response. S1 Table lists the key stakeholders at local, state, and federal levels involved in the response to drinking water contamination emergencies. It specifies their responsibilities and actions, detailing the notification process, their collaboration partners, and their roles in managing the crisis.
(DOCX)

S2 Table Key Information Exchanged in the Game. S2 Table outlines the key information exchanged among the five roles during the City of Leaf Game, detailing their sources and the resulting response actions.
(DOCX)

S3 Table Communication Between Roles in Game Sessions. S3 Table presents the communication interactions between roles across seven game sessions. It shows whether two roles communicated with each other in each group and each round. SC refers to SynthoChem Corporation, R refers to the Resident in the City of Leaf, WTP refers to the Leaf Drinking Water Treatment Plant, EA refers to the Environmental Agency, and HD refers to the Health Department. 1 indicates that communication occurred, while 0 indicates that no communication took place.
(DOCX)

S1 Appendix Serious Game Design Feedback Survey. This survey is used to collect participant feedback from the second game test to identify areas for improvement. The focus of the survey is on gameplay experience, the game’s realism, playability, and flow.
(DOCX)
