# Supplementary material for: Gamifying water crisis management: A serious game for drinking water contamination emergency response
Source: PLoS One. 2025 Apr 1;20(4):e0321210. doi: 10.1371/journal.pone.0321210 (PMC11960903; doi:10.1371/journal.pone.0321210)
Supplement: S1 Table — lists the key stakeholders at local, state, and federal levels involved in the response to drinking water contamination emergencies. It specifies their responsibilities and actions, detailing the notification process, their collaboration partners, and their roles in managing the crisis. (DOCX) [file pone.0321210.s001.docx]

**S1 Table. Stakeholders in Drinking Water Contamination Emergency Response**

S1 Table lists the key stakeholders at local, state, and federal levels involved in the response to drinking water contamination emergencies. It specifies their responsibilities and actions, detailing the notification process, their collaboration partners, and their roles in managing the crisis.

| Roles | Local/State/Federal | Short description about responsibilities/actions |
| --- | --- | --- |
| Responsible Party (Polluter) | Local | If the hazardous spill enters water, the responsible party is required to report immediately to the National Response Center (NRC) [1–4]. In addition, the responsible party is required to provide immediate verbal notification to the State Emergency Management Agency (EMA) [3,5] of any significant release, or threatened release of hazardous materials. The responsible party is liable for costs of containment, cleanup, and damages resulting from the release [6]. |
| 911 | Local | Once 911 is contacted, the 911 dispatcher should prioritize alerting the fire department [7]. Then, the 911 dispatcher should obtain key information such as the incident’s location, time, chemical involved, estimated quantity released, and media into which the release occurred, etc. [7]. Then, the dispatcher should dispatch the appropriate emergency response agencies to the scene. 911 should make sure the local fire department and police department are notified [1]. If the spill could impact the traveling public, State Department of Transportation (DOT) should be notified [5]. |
| Local Fire Department | Local | The local fire department will be the first on the scene at the incident, and is tasked with identifying, rescuing, and mitigating chemical spills (City of Big Rapids, 2021).Also, it will coordinate with the water utility if water service in a specific response area should be shut down, notify affected neighborhoods through methods such as reverse 911 and social media [9], and assist in distributing alternate drinking water supply [9]. |
| Local Police Department | Local | After arrival on scene, local police department will provide traffic control and restrict access to the spill site [9,10]. Also, local police department is responsible for law enforcement (expect on State highways) [10], and may serve as a conduit to state and federal law enforcement and intelligence agencies [9]. In addition, local police department may assist in the distribution of an alternate drinking water supply [9]. |
| Local Department of Health (Local DOH) | Local | Local DOH will declare a health emergency when there is a credible evidence that a release poses an immediate threat to public health [10]. After declaring a health emergency, the local DOH may require relevant parties to provide additional information related to the released hazardous material. Also, they will assist in diagnosing and investigating disease outbreaks, potentially using their own surveillance systems. They may coordinate local healthcare entities and provide mass prophylaxis to prevent epidemics. The local DOH may also collaborate with water utilities in developing public notifications [11]. It also serves as a technical resource during the investigation, providing information about health risks associated with suspected contaminants [9]. |
| Water Utility | Local | Water utility need to contain contaminated water via valves, and restore potable water supply through local alternate source, interconnections, vendors, or emergency treatment. It may need to consult with health authorities, such as local health authorities and water testing labs, if necessary. In addition, it should notify the public of any water advisories [12] |
| Neighboring Water Utility | Local | If the affected water utility has a partnership with neighboring water utilities, supplies, manpower or other resources can be borrowed or shared [8] |
| Local National Guard Units | Local | Local National Guard Units can aid in cordoning off quarantined or contaminated areas and help with alternate water supply acquisition and distribution [13]. |
| Local Emergency Medical Care | Local | Local emergency medical care providers, both in the public and private sectors, are responsible for providing care and transportation to victims of contamination. [10]. |
| Poison Control Center | Local | The Poison Control Center could help with evaluating, assessing and medically managing health exposures associated with hazardous materials spills. It offers a 24/7 toll-free hotline and serves as an information source for the general public, hospital staff, and emergency response personnel [10]. |
| The City | Local | The city need to communicate with residents regarding the impact of the incident on the community, actions taken to protect the public, and updates on the progression of response and recovery efforts [9]. |
| Local Emergency Management Agency | Local | Local Emergency Management Agency will request emergency declaration from the governor, and collaborate with non-municipal water providers to identify distribution locations [12]. |
| State Department of Justice (DOJ) | State | DOJ represents most State agencies in civil litigation arising from hazardous materials incidents, and has general supervisory and enforcement powers under criminal statutes. During such incidents, the DOJ may also assist in criminal intelligence, evidence gathering and analysis, provide surveillance, communications equipment, forensic services, and provide legal advice to state agencies, as necessary [10]. |
| State Emergency Management Agency (EMA) | State | State and local agencies are required to make immediate notification to EMA when responding to an oil spill. State agencies must make verbal notification to EMA for significant emergency situations. For both threatened and actual unauthorized releases of a hazardous substance to water of the state, EMA will notify the appropriate Regional Water Quality Control Board (RWQCB), local health officer, and the administrator of environmental health [10]. |
| State Environmental Protection Agency (State EPA) | State | State EPA can provide recovery assistance following major disasters, including assistance with public health declarations, hazmat identification and removal, and debris management [10]. |
| State Department of Health (State DOH) | State | In the event of a hazardous materials spill or threatened release which affects a public water system or source of drinking water such as a lake, river, or aqueduct, State DOH will work with the water utility to prevent contamination of the system. It will also issue recommendations to the public in coordination with the utility and local health department to prevent use of contaminated water, and provide laboratory support for analysis of drinking water samples[10]. |
| State National Guard | State | State National Guard provides personnel trained in a formal command structure who can perform a wide range of tasks, including distributing bottled-water, collecting samples, staffing call centers, analyzing samples [14]. |
| State Governors | State | State governors have a role in establishing formal agreements with state partners or coordinating funding resources. They should be informed and engaged once contamination has been confirmed to assist in coordination of resources and communication [13]. |
| State Drinking Water And Wastewater Primacy Agencies | State | In a contamination scenario, the primary agency may address regulatory issues related to water use, public notification, environmental concerns, alternative supply quality, and other matters. They may also collaborate with the EPA on remediation and recovery plans. They provide guidance and oversight on public notifications during a water contamination emergency, ensuring that the affected population is informed about the situation and any necessary precautions. They work with the state and water utilities to develop and implement emergency response plans (ERPs) that address water emergencies and are consistent with state and federal regulations [11]. |
| Water Agency Response Network (WARN) | State | WARN members can share resources and personnel across utilities and jurisdictions to ensure that affected communities have access to safe drinking water. For example, if one utility is unable to treat water due to contamination, another utility can step in and provide water (City of Big Rapids, 2021). |
| National Response Center (NRC) | Federal | The NRC, operated by the U.S. Coast Guard, receives reports required on hazardous material spills. Upon notification of an incident, the NRC will immediately notify the appropriate Federal On-Scene Coordinator (FOSC) and concerned Federal agencies. Federal law mandates immediate notification to the NRC whenever a reportable quantity of a hazardous material is released into the environment. If uncertain about whether the amount released meets the required reporting levels for these materials, the NRC should be notified [1]. |
| United States Environmental Protection Agency (USEPA) | Federal | USEPA designates a FOSC for incidents within its jurisdiction, with access to federal funding for containment and mitigation of releases. The FOSC has the authority to mobilize Special Forces and Technical Support Centers to support major spill response and cleanup efforts [10]. |
| United States Department of Health and Human Services (DHHS) | Federal | DHHS makes determinations that illness, disease, or complaints that may be attributable to exposure to a hazardous substance, pollutant, or contaminant. DHHS also provides expert advice and assistance on actual or potential discharges or releases that pose a threat to public safety and health [10]. |
| Agency for Toxic Substances and Disease Registry (ATSDR) | Federal | ATSDR could assist in assessing public health threats posed by an incident, provide advice on the adequacy of personnel protection measures within the response area, investigate health complaints, provide advice on potential relocation of nearby residents, and coordinate the appropriate health response with public health agencies and the private medical community [10]. |
| Centers For Disease Control and Prevention (CDC) | Federal | CDC operates a network of laboratories, including the Laboratory Response Network (LRN), which supports the detection, identification, and confirmation of waterborne contaminants, including biological, chemical, and radiological agents. CDC assesses the public health risks associated with water contamination incidents and helps determine appropriate protective actions. CDC develops and disseminates public health messages, guidelines, and recommendations to inform and educate the public, healthcare providers, and other stakeholders about water contamination incidents and associated health risks [11]. |
| United States Department of Homeland Security (DHS) | Federal | DHS will work with the FBI to establish and operate a JIC in the field as the focal point for information to the public and the media concerning the federal response to the emergency. As needed, DHS can provide resources from the National Disaster Medical System and/or the Metropolitan Medical Response System [15]. |
| United States Coast Guard (USCG) | Federal | The USCG operates the NRC and maintains a capability to contain and clean up polluting substances in waters and on shores within their jurisdiction through the National Strike Force (NSF). The USCG will provide the FOSC for incidents within their jurisdiction and can access federal funding for abating and mitigating releases. Responsibility for long-term removal actions may be transferred to US EPA. The FOSC will use appropriate legislative and regulatory authorities, the NCP, area plans, regional and local contingency plans, and other circumstances unique to each incident to ensure that pollution response is carried out expeditiously and aggressively [10]. |
| Federal Emergency Management Agency (FEMA) | Federal | FEMA will mobilize federal response within 3 to 5 days after the event. FEMA will also obtain bottled water and deliver water to state distribution sites [12] |
| United States Army Corps of Engineers (USACE) | Federal | USACE assists in the provision of temporary restoration of water supplies and emergency contracting [13]. |
| Response & Cleanup Companies | Others | If no public hazardous materials emergency response team is available, managing initial contamination containment may necessitate hiring response and cleanup companies. These companies provide services under contract and have the capability to clean up, haul, and decontaminate a hazardous materials incident scene, as well as conduct restoration and repair of highways or other damaged property [10]. |
| Environmental and Public Health Laboratories | Others | Environmental and public health laboratories will provide or coordinate laboratory support for the analysis of water samples during investigation and remediation efforts [16]. |
| Water Vendors | Others | Water vendors can provide the necessary emergency water supplies [8]. |
| Non-Governmental Organizations (NGOs) | Others | NGOs, such as The Red Cross and the Salvation Army, can provide a range of supports, such as disaster relief and humanitarian aid, health and medical support, and community engagement [13]. |
| Residents | Others | Residents can report unusual conditions or changes in water quality (e.g., taste, color, odor) to water utilities [16], local officials such as fire or police departments [17], state agencies or federal agencies such as USEPA[18]. They can contact local DOH or a state certified laboratory to perform water test [16]. They have the right to sue the polluter or any regulatory agency for their failure to enforce the Clean Water Act [19]. |

**References**

1. PHMSA. 2020 Emergency Response Guidebook. 2020. Available: http://www.sct.gob.mxhttps://www.phmsa.dot.gov/hazmathttps://www.tc.gc.ca/TDG

2. USEPA. United States Environmental Protection Agency, How to Report Spills and Environmental Violations. 2022 [cited 23 Mar 2023]. Available: https://www.epa.gov/pesticide-incidents/how-report-spills-and-environmental-violations

3. EcologyWA. Report a spill. 2023 [cited 23 Mar 2023]. Available: https://ecology.wa.gov/Footer/Report-an-environmental-issue/Report-a-spill

4. NHDES. New Hampshire Department of Environmental Services, Report a Spill. 2023 [cited 23 Mar 2023]. Available: https://www.des.nh.gov/waste/spill-response/report-spill

5. WSDOT. Report a spill. 2023 [cited 27 Mar 2023]. Available: https://wsdot.wa.gov/construction-planning/protecting-environment/report-spill

6. USEPA. Who Pays. 2023 [cited 26 Jun 2024]. Available: https://www.epa.gov/emergency-response/who-pays#:~:text=By%20law%2C%20the%20parties%20responsible,related%20to%20their%20own%20activities.

7. Lagrange County LEPC. Lagrange County Local Emergency Planning Committee Hazardous Materials Emergency Response Plan. 2022.

8. City of Big Rapids. Water System Emergency Response Plan. 2021. Available: https://cms6.revize.com/revize/bigrapidsmi/document_center/Public%20Works/Emergency%20responce%20plan..pdf

9. USEPA. United States Environmental Protection Agency, Guidance for Responding to Drinking Water Contamination Incidents Disclaimer. 2018.

10. Cal OES. California Governor’s Office of Emergency Services, HazMat Tool Kit Part 3: Roles and Responsibilities. 2011. Available: http://www.calcupa.net

11. USEPA. Response Protocol Toolbox: Planning for and Responding to Drinking Water Contamination Threats and Incidents, Module 5: Public Health Response Guide for Drinking Water Utilities. 2004. Available: https://www.epa.gov/waterutilityresponse/module-5-public-health-response-guide-drinking-water-utilities

12. Salus Resilience. Emergency Drinking Water Framework. 2022. Available: https://www.regionalh2o.org/sites/default/files/2022-1130%20Final%20Emergency%20Drinking%20Water%20Framework-F-FULL%20REPORT.pdf

13. USEPA. United States Environmental Protection Agency, Planning for an Emergency Drinking Water Supply. EPA 600/R-11/054. 2011; 1–40. Available: https://www.epa.gov/sites/production/files/2015-03/documents/planning_for_an_emergency_drinking_water_supply.pdf

14. USEPA. Guidance for Responding to Drinking Water Contamination Incidents. 2018. Available: https://nepis.epa.gov/Exe/ZyNET.exe/P100VVRC.TXT?ZyActionD=ZyDocument&Client=EPA&Index=2016+Thru+2020&Docs=&Query=&Time=&EndTime=&SearchMethod=1&TocRestrict=n&Toc=&TocEntry=&QField=&QFieldYear=&QFieldMonth=&QFieldDay=&IntQFieldOp=0&ExtQFieldOp=0&XmlQuery=&File=D%3A%5Czyfiles%5CIndex%20Data%5C16thru20%5CTxt%5C00000010%5CP100VVRC.txt&User=ANONYMOUS&Password=anonymous&SortMethod=h%7C-&MaximumDocuments=1&FuzzyDegree=0&ImageQuality=r75g8/r75g8/x150y150g16/i425&Display=hpfr&DefSeekPage=x&SearchBack=ZyActionL&Back=ZyActionS&BackDesc=Results%20page&MaximumPages=1&ZyEntry=1&SeekPage=x&ZyPURL

15. USEPA. United States Environmental Protection Agency, Response Protocol Toolbox: Planning for and Responding to Drinking Water Contamination Threats and Incidents Module 2: Contamination Threat Management Guide. 2003.

16. CDC. Drinking Water Frequently Asked Questions (FAQs). 2023 [cited 26 Jun 2024]. Available: https://www.cdc.gov/healthywater/drinking/drinking-water-faq.html

17. NHDES. Complaints. 2024 [cited 26 Jun 2024]. Available: https://www.des.nh.gov/complaints#:~:text=First%3A%20Contact%20local%20officials%20such,submit%20the%20SRCIS%20complaint%20form.

18. USEPA. Report an Environmental Violation, General Information. 2024 [cited 26 Jun 2024]. Available: https://www.epa.gov/enforcement/report-environmental-violation-general-information

19. OEC. Ohio Environmental Council, Guide to Clean Water Act Citizen Suits. 2010. Available: https://www.waterboards.ca.gov/water_issues/programs/swamp/docs/cwt/guidance/112a1.pdf
