# Supplementary material for: Gamifying water crisis management: A serious game for drinking water contamination emergency response
Source: PLoS One. 2025 Apr 1;20(4):e0321210. doi: 10.1371/journal.pone.0321210 (PMC11960903; doi:10.1371/journal.pone.0321210)
Supplement: S2 Table — outlines the key information exchanged among the five roles during the City of Leaf Game, detailing their sources and the resulting response actions. (DOCX) [file pone.0321210.s002.docx]

**S2 Table. Key Information Exchanged in the Game**

S2 Table outlines the key information exchanged among the five roles during the City of Leaf Game, detailing their sources and the resulting response actions.

| Key Information | Source(s) | Result(s) |
| --- | --- | --- |
| Initial Awareness of Contamination | - Residents (report water issues) - SynthoChem Corporation (disclose spill) | Initiates initial investigation including water quality test. |
| Identification of the Contaminant (TXC) | - SynthoChem Corporation (disclose the spilled chemical) - Leaf Drinking Water Treatment Plant (conduct water test) - Environmental Agency (conduct water test) | Allows target response for the identified contaminant, TXC, including identifying the TXC manufacturer, and conducting specific health and toxicity tests. |
| Treatment Standards for TXC | - Health Department (conduct toxicity analysis) | Establishes a temporary Maximum Contaminant Level (MCL) for TXC, guiding Leaf Drinking Water Treatment Plant’s water supply strategies. |
| TXC Concentration Changes Over Rounds | - Water Treatment Plant (conduct continuous water tests) - Environmental Agency (use predictive model to track contamination changes over time) | Provides ongoing updates on contaminant levels, helping to shape Leaf Drinking Water Treatment Plant’s water supply strategies and forecast the timeline for resolving the water crisis. |
