# Supplementary material for: Gamifying water crisis management: A serious game for drinking water contamination emergency response
Source: PLoS One. 2025 Apr 1;20(4):e0321210. doi: 10.1371/journal.pone.0321210 (PMC11960903; doi:10.1371/journal.pone.0321210)
Supplement: S3 Table — presents the communication interactions between roles across seven game sessions. It shows whether two roles communicated with each other in each group and each round. SC refers to SynthoChem Corporation, R refers to the Resident in the City of Leaf, WTP refers to the Leaf Drinking Water Treatment Plant, EA refers to the Environmental Agency, and HD refers to the Health Department. 1 indicates that communication occurred, while 0 indicates that no communication took place. (DOCX) [file pone.0321210.s003.docx]

**S3 Table. Communication Between Roles in Game Sessions**

S3 Table presents the communication interactions between roles across seven game sessions. It shows whether two roles communicated with each other in each group and each round. SC refers to SynthoChem Corporation, R refers to the Resident in the City of Leaf, WTP refers to the Leaf Drinking Water Treatment Plant, EA refers to the Environmental Agency, and HD refers to the Health Department. 1 indicates that communication occurred, while 0 indicates that no communication took place.

| Group # | Round # | SC-R | SC-WTP | SC-EA | SC-HD | R-WTP | R-EA | R-HD | WTP-EA | WTP-HD | EA-HD |
| --- | --- | --- | --- | --- | --- | --- | --- | --- | --- | --- | --- |
| 1 | 1 | 0 | 1 | 1 | 0 | 1 | 0 | 1 | 1 | 0 | 1 |
|  | 2 | 0 | 0 | 1 | 1 | 1 | 0 | 1 | 1 | 1 | 1 |
|  | 3 | 0 | 0 | 1 | 0 | 1 | 0 | 1 | 1 | 1 | 1 |
|  | 4 | 1 | 1 | 1 | 1 | 0 | 1 | 1 | 1 | 0 | 1 |
|  | 5 | 0 | 0 | 0 | 0 | 1 | 1 | 0 | 1 | 0 | 0 |
|  | 6 | 0 | 0 | 0 | 0 | 0 | 0 | 0 | 1 | 0 | 0 |
| 2 | 1 | 1 | 1 | 0 | 0 | 1 | 0 | 0 | 0 | 0 | 1 |
|  | 2 | 1 | 1 | 0 | 1 | 0 | 0 | 1 | 1 | 0 | 0 |
|  | 3 | 1 | 1 | 0 | 1 | 0 | 1 | 1 | 0 | 1 | 0 |
|  | 4 | 1 | 1 | 1 | 1 | 0 | 0 | 1 | 0 | 0 | 1 |
|  | 5 | 1 | 1 | 1 | 1 | 1 | 0 | 1 | 1 | 1 | 0 |
|  | 6 | 1 | 1 | 1 | 1 | 1 | 1 | 1 | 0 | 0 | 0 |
|  | 7 | 1 | 1 | 1 | 1 | 0 | 1 | 1 | 1 | 0 | 1 |
| 3 | 1 | 0 | 0 | 1 | 1 | 1 | 1 | 1 | 1 | 1 | 1 |
|  | 2 | 1 | 1 | 1 | 1 | 1 | 1 | 1 | 1 | 1 | 1 |
|  | 3 | 1 | 1 | 1 | 0 | 1 | 1 | 0 | 1 | 1 | 1 |
|  | 4 | 1 | 1 | 1 | 1 | 1 | 1 | 1 | 1 | 1 | 0 |
| 4 | 1 | 1 | 1 | 0 | 0 | 1 | 1 | 1 | 1 | 1 | 0 |
|  | 2 | 1 | 0 | 1 | 1 | 1 | 0 | 1 | 1 | 1 | 1 |
|  | 3 | 0 | 1 | 0 | 1 | 0 | 1 | 1 | 1 | 1 | 0 |
|  | 4 | 1 | 1 | 1 | 0 | 1 | 1 | 1 | 1 | 1 | 0 |
|  | 5 | 1 | 1 | 1 | 1 | 1 | 1 | 1 | 1 | 1 | 1 |
| 5 | 1 | 0 | 1 | 0 | 0 | 1 | 1 | 0 | 0 | 0 | 1 |
|  | 2 | 0 | 0 | 0 | 1 | 1 | 0 | 1 | 1 | 0 | 1 |
|  | 3 | 0 | 0 | 1 | 0 | 1 | 0 | 1 | 1 | 0 | 1 |
|  | 4 | 0 | 0 | 0 | 0 | 1 | 0 | 1 | 1 | 1 | 1 |
|  | 5 | 0 | 1 | 0 | 1 | 1 | 0 | 1 | 1 | 0 | 1 |
|  | 6 | 0 | 0 | 0 | 1 | 1 | 1 | 0 | 1 | 0 | 0 |
| 6 | 1 | 1 | 0 | 0 | 0 | 1 | 0 | 0 | 0 | 0 | 1 |
|  | 2 | 0 | 1 | 1 | 1 | 1 | 0 | 1 | 0 | 1 | 0 |
|  | 3 | 0 | 1 | 1 | 1 | 0 | 0 | 1 | 1 | 0 | 1 |
|  | 4 | 1 | 0 | 1 | 0 | 0 | 0 | 0 | 0 | 1 | 0 |
|  | 5 | 1 | 1 | 0 | 0 | 0 | 0 | 0 | 1 | 0 | 0 |
| 7 | 1 | 1 | 1 | 0 | 0 | 1 | 0 | 0 | 1 | 0 | 0 |
|  | 2 | 1 | 1 | 0 | 0 | 1 | 0 | 1 | 1 | 1 | 1 |
|  | 3 | 1 | 0 | 1 | 1 | 0 | 0 | 1 | 1 | 1 | 1 |
|  | 4 | 1 | 0 | 0 | 0 | 1 | 0 | 0 | 0 | 0 | 0 |
|  | 5 | 0 | 0 | 1 | 0 | 1 | 0 | 0 | 1 | 1 | 1 |
|  | 6 | 1 | 0 | 0 | 0 | 0 | 0 | 0 | 1 | 1 | 0 |
|  | 7 | 0 | 0 | 0 | 0 | 1 | 0 | 0 | 1 | 1 | 0 |
