# Supplementary material for: Gamifying water crisis management: A serious game for drinking water contamination emergency response
Source: PLoS One. 2025 Apr 1;20(4):e0321210. doi: 10.1371/journal.pone.0321210 (PMC11960903; doi:10.1371/journal.pone.0321210)
Supplement: S1 Appendix — This survey is used to collect participant feedback from the second game test to identify areas for improvement. The focus of the survey is on gameplay experience, the game’s realism, playability, and flow. (DOCX) [file pone.0321210.s004.docx]

**S1 Appendix. Serious Game Design Feedback Survey**

This survey is used to collect participant feedback from the second game test to identify areas for improvement. The focus of the survey is on gameplay experience, the game’s realism, playability, and flow.

Thank you for being a part of our gaming experience! Your detailed and honest feedback is vital in helping us enhance gameplay for future sessions.

This survey should take about 10-15 minutes to complete. We greatly appreciate your time and assistance in making the game better.

To better understand your feedback, we would first like to know the role you played in the game. Your role perspective will help us to fine-tune specific elements of the game more adeptly.

Q1. What role did you play in the game?

1. SynthoChem Corporation
2. Resident in the City of Leaf
3. Leaf Drinking Water Treatment Plant
4. Environmental Agency
5. Health Department

Please take a moment to reflect on your individual experience and provide us with your honest feedback through the following questions. We appreciate your time and insights.

Q2. Did you find the game instructions clear and easy to follow? If not, what areas would benefit from further clarification?

Q3. Would you need more background information or context at any point during the game?

Q4. Were there any aspects of the game's rules or mechanics that you found confusing or inconsistent?

Q5. Was there a balance between the complexity of the game and its playability to players?

Q6. Were there any aspects of the game that felt particularly realistic or unrealistic?

Q7. Did you feel that the game encouraged active participation from all players?

Q8. Would you suggest any adjustments to the role dynamics or the rules to enhance the gameplay experience?

Q9. Do you have any other suggestions or comments about the game design?

We thank you for your time spent taking this survey.

Your response has been recorded.
